# Maternofetal consequences of *Coxiella burnetii* infection in pregnancy: a case series of two outbreaks

**DOI:** 10.1186/1471-2334-12-359

**Published:** 2012-12-19

**Authors:** Katharina Boden, Andreas Brueckmann, Christiane Wagner-Wiening, Beate Hermann, Klaus Henning, Thomas Junghanss, Thomas Seidel, Michael Baier, Eberhard Straube, Dirk Theegarten

**Affiliations:** 1 Institute of Clinical Chemistry and Laboratory Medicine, University Hospital Jena, Erlanger Allee 101, 07747, Jena, Germany; 2 Department of Gynecology and Obstetric, University Hospital, Jena, Bachstraße 18, 07743, Jena, Germany; 3 Q fever Consulting Laboratory, Baden-Wuerttemberg, State Health Office, Nordbahnhofstraße 135, 70191, Stuttgart, Germany; 4 Institute of Medical Microbiology, University Hospital Jena, Erlanger Allee 101, 07747, Jena, Germany; 5 Institute of Epidemiology, Friedrich-Loeffler-Institute, Seestraße 55, 16868, Wusterhausen, Germany; 6 Section of Clinical Tropical Medicine, University Hospital Heidelberg, Im Neuenheimer Feld 324, 69120, Heidelberg, Germany; 7 Department of Gastroenterology, Hepatology and Infectious Diseases, University Hospital Jena, Erlanger Allee 101, 07747, Jena, Germany; 8 Institute of Pathology and Neuropathology, University Hospital Essen, University Duisburg-Essen, Hufelandstrasse 55, 45147, Essen, Germany

## Abstract

**Background:**

A high complication rate of Q fever in pregnancy is described on the basis of a limited number of cases. All pregnant women with proven Q fever regardless of clinical symptoms should therefore receive long-term cotrimoxazole therapy. But cotrimoxazole as a folic acid antagonist may cause harm to the fetus. We therefore investigated the Q fever outbreaks, Soest in 2003 and Jena in 2005, to determine the maternofetal consequences of *Coxiella burnetii* infection contracted during pregnancy.

**Methods:**

Different outbreak investigation strategies were employed at the two sides. Antibody screening was performed with an indirect immunofluorescence test. Medical history and clinical data were obtained and serological follow up performed at delivery. Available placental tissue, amniotic fluid and colostrum/milk were further investigated by polymerase chain reaction and by culture.

**Results:**

11 pregnant women from Soest (screening rate: 49%) and 82 pregnant women from Jena (screening rate: 27%) participated in the outbreak investigation. 11 pregnant women with an acute *C. burnetii* infection were diagnosed. Three women had symptomatic disease.

Three women, who were infected in the first trimester, were put on long-term therapy. The remaining women received cotrimoxazole to a lesser extent (n=3), were treated with macrolides for three weeks (n=1) or after delivery (n=1), were given no treatment at all (n=2) or received antibiotics ineffective for Q fever (n=1). One woman and her foetus died of an underlying disease not related to Q fever. One woman delivered prematurely (35^th^ week) and one child was born with syndactyly. We found no obvious association between *C. burnetii* infection and negative pregnancy outcome.

**Conclusions:**

Our data do not support the general recommendation of long-term cotrimoxazole treatment for Q fever infection in pregnancy. Pregnant women with symptomatic *C. burnetii* infections and with chronic Q fever should be treated. The risk-benefit ratio of treatment in these patients, however, remains uncertain. If cotrimoxazole is administered, folinic acid has to be added.

## Background

*Coxiella burnetii*, an obligatory intracellular bacterium, is the causative agent of Q fever, a zoonotic disease with worldwide distribution. The clinical manifestations of acute Q fever appear as atypical pneumonia, systemic febrile illness or acute hepatitis. Roughly 50% of all infections with *C. burnetii* are asymptomatic [1].

Q fever is diagnosed by detection of antibodies against two antigenic variations of the *C. burnetii* lipopolysaccharide. IgM- and IgG-antibodies mainly directed against the truncated form of lipopolysaccharide, called Phase II (Ph2), appear in acute infection. In chronic Q fever, high levels of IgG antibodies directed against Phase I (Ph1), the complete lipopolysaccharide, are detectable. Isolation of *C. burnetii* from medical specimen is difficult as isolation is time-consuming and requires a biosafety level 3 laboratory. Thus, detection by polymerase chain reaction (PCR) became a useful additional tool in the past years [2,3].

Abortion material and birth products from ruminants are the most commonly identified sources of human infections. In these animals *C. burnetii* is associated with abortions.

The pathogenic role of *C. burnetii* in pregnant women is still uncertain. As of 2007, only 74 cases appeared in publication. Of these some had serious complications such us intrauterine fetal death (IUFD), maternofetal death and spontaneous abortions. Based on these 74 cases long-term cotrimoxazole therapy of at least five weeks duration has been recommended [4].

In Germany, Q fever outbreaks happen sporadically. About 40 outbreaks in humans are documented from 1947 to 1999 [5].

Two significant outbreaks in Germany occured in Soest in 2003 and in Jena in 2005.

The outbreak Soest was caused by a lambing sheep at a farmers market that took place on May 3 and 4 in 2003 in a spa town near Soest. Approximately 3,000 visitors from different parts of Germany visited the market. A local hospital informed the health department of Soest of an increase of atypical pneumonia 23 days later, May 26 2003. Altogether 299 cases related to this outbreak were reported [6].

In Jena, from June 2^nd^ to 18th 2005, 300 ewes with 35 lambing were grazing near densely populated area of 11,500 inhabitants. On the 27th of June, a practitioner informed the health authorities of an increased number of pneumonia in this district (Winzerla). The flock of sheep was promptly identified as a potential source. Suspected Q fever was confirmed few days later in animals and humans [7]. Within a period of seven weeks (13 June – 24 July), 331 cases were reported [8].

At both sites, Soest and Jena screening programs were implemented to identify people with special risk for severe disease; e.g., pregnancy.

We analyzed the obtained data to evaluate the maternofetal consequences of *Coxiella burnetii* infection contracted during pregnancy.

## Results

### Screening coverage

**Figure 1:**
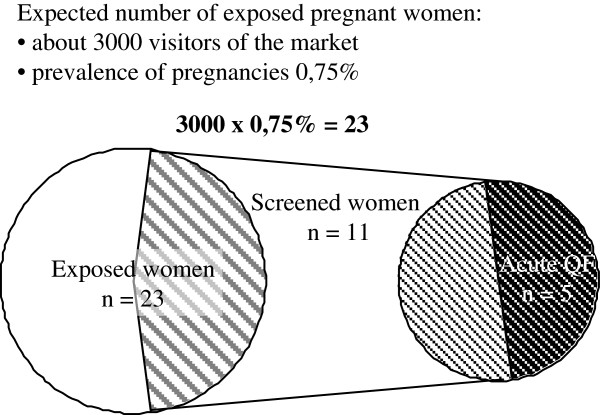
Screening for pregnant women exposed at the farmers market Soest.

Eleven pregnant women exposed at the farmers market Soest took part in the screening program. This corresponds to a screening rate of 49% (Figure 1).

**Figure 2:**
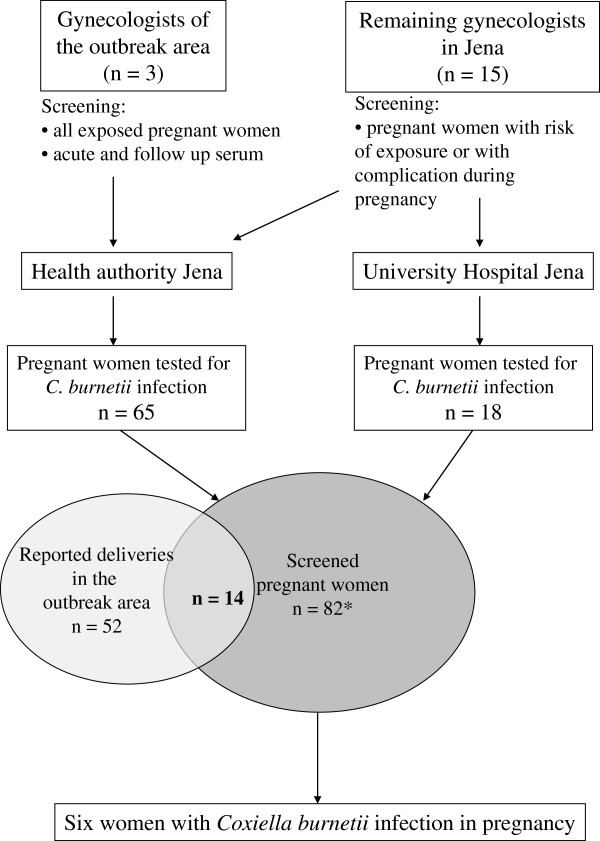
Screening for pregnant women exposed at the outbreak in Jena (*one woman was double tested).

82 pregnant women were included in the screening program in Jena. 52 childbirths were registered in the outbreak area during the 9 months following the exposure. Out of these, 14 childbirths were covered by our screening program, corresponding to a screening rate of 27% (Figure 2).

### Women with *C. burnetii* infection in pregnancy

Altogether eleven pregnant women with an acute *C. burnetii* infection were diagnosed, five in Soest in 2003 and six in Jena in 2005. Four of the five women in Soest, have been briefly discussed [6]. The characteristics of the 11 patients are presented in Table 1. Three women had symptomatic disease, presenting with fever; one also presented with pneumonia. All women seroconverted during pregnancy.

**Table 1:** **Pregnant women with *C. burnetii *infection contracted during pregnancy.**

**Trimester of Exposure**	**Cases [n]**	**Clinical signs of Q fever (n)**	**antibiotic treatment (n)**	**Gestational week at delivery**	**Condition of the infant (n)**	**PCR on placenta positive**	**culture on placenta positive**	**PCR on colostrum/ milk positive**	**PCR on amniotic fluid positive**	**Ph1-IgG > 1:800**
1	3*	fever (1), none (2)	Until delivery:	40	syndactyly^2^ (1), well^3^ (1)	0/2	0/1	0/1	0/2	1
			· Clarithromycin (1)							
			· Trimethoprim-Sulfamethoxazol (1)							
			· (Sulfadiazin + Pyrimethamin for 2 weeks); Trimethoprim-Sulfamethoxazol + Pyrimethamin ^1^ (1)							

2	4	none (4)	· Trimethoprim-Sulfamethoxazol for one week (2)	40	RAD (1), well (3)	0/4	0/3	1/3	0/3	1
			· Clarithromycin after delivery (1)							
			· Trimethoprim-Sulfamethoxazol for four weeks (1)							
3	4	pneumonia**(1), fever (1), none (2)	· Erythromycin/Clarithromycin for three weeks (1)	35-40	RDS (1), well (3), Oligoamnios (1)	0/2	0/2	0/1	0/1^4^	0
			· without (2)							
			· Amoxicillin followed by Imipenem (1)							

NOTE. * maternofetal death caused by an underlying disease other than Q fever (n=1), ** confirmed by Xray; ^1^ additional Toxoplasmose infection in pregnancy; ^2^toes II-III; ^3^ no C. burn-specific IgM-antibodies; ^4^ additional PCR on cord blood negative; RAD, respiratory adaption disorder; RDS, respiratory distress syndrome.

No severe obstetrical complications; such as spontaneous abortion, intrauterine fetal death or maternofetal death, occurred due to Q fever. One woman, however, acquired the infection in week six, recovered without treatment but was soon put under cotrimoxazole treatment for prophylaxis. Because of pre-existing congestive heart failure with the risk of decompensation during pregnancy she had to undergo valvuloplasty two months later. Unfortunately she developed postoperative complications and both she and her foetus died. The maternal serum Ph1 IgG antibody level was not elevated. The placenta did not show signs of placentitis on microscopy.

All other women delivered full term (week 39-40, birth weight 2970-4040 g) except one, who developed pneumonia at week 27 and delivered a baby (3250 g) at week 35. Other findings were oligohydramnion close to delivery (one case) and one child with a syndactyly of toe II-III at birth. Her mother had received clarithromycin treatment until delivery.

5 out of 7 placentas were investigated by PCR and culture and two by PCR alone. All were negative. The PCR on colostrum/breast milk was positive in one out of five samples investigated and breastfeeding was stopped. The child had an uneventful follow up, whereas the mother revealed a serological antibody pattern compatible with chronic infection without echocardiographic signs or symptoms of disease.

PCR performed on amniotic fluid was negative. A serological profile compatible with chronic Q fever was found in two women. No clinical or echocardiographic signs of endocarditis were detected.

Antibiotic treatment was administered in nine cases (Table 1). The recommended long-term cotrimoxazole therapy was given to two women and one woman received clarithromycin up to delivery. All three women were infected in the first trimester. As noted above one died of an underlying disease unrelated to Q fever. One had additional infection with *Toxoplasma gondii* but delivered a full term healthy baby. The third woman, who was treated with clarithromycin gave birth to a newborn with syndactyly of the right foot (dig II-III).

The other eight of eleven women were not treated with the recommended cotrimoxazole therapy of at least five weeks [4]. One woman was treated for Q fever pneumonia with parenteral antibiotics (Erythromycin/Clarithromycin) for three weeks. She prematurely delivered a 3250 g healthy baby in week 35. At the time of birth she was healthy and all relevant tests were negative for *C. burnetii*. All other women delivered full term without complications.

## Discussion

The estimated screening rate was very good in the outbreak Soest, although the visitors to the farmers market came from various locations in Germany. Conversely, the screening rate in Jena was only 27%. It is important to point out, however, that the screening rate in Soest was calculated using an estimated number of pregnant women exposed. The low screening rate in Jena was probably due to the fact that the information letter did not contain sufficient details regarding the expected benefits of screening and treatment. A recent study on determinants for refusing participation on Q fever screening in pregnancy found a response rate of 56%. Approximately one quarter refused to participate because they had doubts about the side effects of the antibiotic treatment or were afraid of the consequences of participation [9]. Another reason for the low screening rate in Jena could have been the misconception that *C. burnetii* infection contracted during pregnancy would always be symptomatic. This, and the frequent request to screen all pregnant women with complications, could have led to a selection of pregnancies with complications or concomitant diseases. Our rate of 27% (3/11) of symptomatic women compared to 10% (1/10) in a previous study corresponds to preselection [10].

However, we found no obvious association between *C. burnetii* infection and negative pregnancy outcome, although 73% (8/11) of the women did not receive the recommended cotrimoxazole therapy of at least five weeks [4]. The woman that was treated with clarithromycin for the entire pregnancy gave birth to a newborn with syndactyly. Syndactyly is a common fetal malformation (approximately 1 of 200 births) and several genetic disorders can cause this disease [11]. Given the high incidence of the disorder and the consequent treatment of the mother, Q fever as causative agent appears to be unlikely but cannot be ruled out.

The woman treated for Q fever pneumonia with erythromycin/clarithromycin for three weeks, prematurely delivered although all relevant tests were negative for *C. burnetii*. Given Germany’s premature birth rate of 7% this prematurity could be coincidental. All other women delivered full term without complications.

In contrast to our findings a study, of 53 pregnant women diagnosed with Q fever at the French National Reference Centre for Rickettsioses revealed obstetric complications in 81% of the 37 women without long-term cotrimoxazole therapy [4]. But the study had a high probability of a bias towards complicated cases.

Published evidence on the association between Q fever and negative pregnancy outcomes is low. A systematic review in 1990 identified only articles with level IV and V evidence [12]. Of the 84 cases reported in the literature to date, 53 were part of the large case series noted above [4,13-16].

A recent large population based study in the Netherlands investigated 1174 serum samples collected by an existing national prenatal screening programme (at the 12^th^ week of pregnancy) and data from the Netherlands Perinatal Registry on diagnosis and outcome. Out of these serum samples, 56 cases with acute *C. burnetii* infection during pregnancy were identified and no association between *C. burnetii* infection and preterm delivery, low birth weight or perinatal mortality was observed [17]. In another comprehensive study with 4588 participants, including 200 seropositive women, they found an association between phase II antibody titre ≥ 1:32 and gestational age ≤ 36 weeks, current or previous neonatal death, and higher parity. *C. burnetii* was not identified by PCR or culture in the placentas investigated [18].

The agent has been isolated in intact as well as necrotic placentas with immaturity, abortion, maternofetal death and stillbirth [4,14,16,19-23], but also from normal placentas in undisturbed pregnancies [21,24-26]. This suggests evidence against *C. burnetii* infection posing a high risk to pregnancies. In our study all examined placentas were negative for *C. burnetii.*

We found *C. burnetii* in the milk of one woman with serological antibody pattern compatible to chronic infection but no clinical signs. Breastfeeding was stopped and the child had an uneventful follow up. In other reports *C. burnetii* was found repeatedly in human milk with unclear implications for the breastfed child [24,27,28].

Whether pregnant women have an increased risk of developing clinically apparent chronic Q fever remains unresolved. In the largest case series published, 28 of 53 pregnant women developed a serological profile of chronic Q fever. Three out of these 28 women developed an endocarditis, corresponding to 7% of all included pregnant women [4]. A follow up study on 1569 acute Q fever cases revealed a development of endocarditis in 12 (0.76%) cases [29]. This suggests a higher risk for women infected during pregnancy compared with the general population. In our study two patients developed a serological profile of chronic Q fever but none developed clinically apparent chronic Q fever. Altogether our limited data cannot yet give conclusive answers to this question.

Several antibiotics such as cotrimoxazole, ciprofloxacine, azithromycin, rifampicin, clarithromycin, doxycycline, erythromycin and tifomycin have been given to treat Q fever in pregnancy. The only study investigating antibiotic treatment of Q fever in pregnancy found that long-term cotrimoxazole therapy prevented obstetric complications (p=0.009). However, patients (n=16) who presented with obstetric complications at the time of diagnoses did not receive the long-term cotrimoxazole therapy. Investigating only the 37 women with no complications at the time of diagnoses, the efficacy of long-term cotrimoxazole therapy to prevent IUFD was less significant (0.047). Nevertheless, administration for all pregnant women with proven Q fever was recommended [4]. Even under cotrimoxazole therapy *C. burnetii* was detected in the placenta in some cases [4,16,25]. Cotrimoxazole is a folic acid antagonist that inhibits deoxyribonucleic acid synthesis by interfering with the production of folic acid. Exposure to it during pregnancy appears to be associated with an increased risk of small-for-gestational-age newborns, preterm births, cardiovascular and neural tube defects [30-33]. Additional folinic acid supplementation has a strong effect in the reduction of preterm birth and defects of the neural tube [32,34].

## Conclusions

We conclude that current knowledge about Q fever in pregnancy is less than adequate. With the limited evidence available to support treatment of pregnant women with cotrimoxazole, and considering the risk of harming the fetus, the recommendation of long-term cotrimoxazole treatment for every pregnant woman with laboratory confirmed Q fever is questionable. On the other hand pregnant women with symptomatic *C. burnetii* infections and with chronic Q fever should be treated. The risk-benefit ratio of treatment in these patients, however, is also not clear. If cotrimoxazole is administered, folinic acid has to be added.

## Methods

### Patients

Two different strategies were used to investigate the outbreaks in Jena and Soest. Most of the visitors in Soest came from various locations in Germany. Because of this an appeal to screen every pregnant woman who had attended the farmers market was published in the Journal of the German Medical Association. The county Health authority used local press and publicized the need to test all pregnant women. The appeal also offered free antibody testing at the Q fever National Consulting Laboratory (NCL). To evaluate the outbreak investigation strategies used in Soest, we first had to estimate the total number of pregnant women who had visited the market. Using the approximate number of visitors (3000) and factoring in the known German birth-rate (1/100/year), 0.75% is the prevalence of pregnant women. This results in an estimate of 23 pregnant women exposed in Soest (Figure 1).

The features of outbreak Jena enabled the local Health Authority and the Robert Koch Institute to start a very intense information policy rapidly. Within one week of confirming the first human case of Q fever all gynaecologists and obstetricians in the town (n=18), the Jena midwives birthing centre and the Department of the University Hospital were notified by a letter which recommended the screening of all women with complicated pregnancies. In the second week the gynaecologists in the affected area (n=3) were encouraged by telephone calls to screen all pregnant women with or without complications. In the third week information letters with the request to screen all pregnant women were sent to all medical practices and also placed at the front doors of all housing units in the affected area. The NCL again offered free testing to exposed people. The maternity clinic of the University Hospital was designated as centre for assisting infected women and the screening was performed by the Health Authority Jena and at the University Hospital Jena (Figure 2).

The registration office in Jena provided a list of women living in the outbreak area and giving birth in the nine month following the possible exposure. To evaluate the coverage of our outbreak investigations we compared this list to our list of screened women in the well-defined outbreak area (Figure 2).

Medical history and clinical data were collected by the responsible gynaecologists, paediatricians, the NCL and the Department of Gastroenterology, Hepatology and Infectious Diseases of the University Hospital Jena. Preterm birth was defined as gestational age of less than 37 completed weeks. A serological follow up was performed at delivery. Specimens of placental tissue and amniotic fluid were obtained whenever possible. Additionally breast milk or/and colostrum from the women exposed in the outbreak in Soest were collected. All patients’ samples were taken as part of standard care. The study was approved by the Ethical committee of the University Hospital Jena (reference number 3439-04/12).

### Serological testing

The serological diagnosis was done by a commercially available indirect immunofluorescence antibody test IFAT (BIOS/Focus, Cypress CA). For the IFAT all sera were tested at dilutions 1:16, 1:64, 1:246 to 1:1024 in phosphate buffered saline (PBS - BIOS, Germany, pH 7.8) and fluorescein isothiocyanate-labelled goat anti-human IgG/IgM antibodies (BIOS, Germany) were used as conjugate. The examination was done by fluorescent microscopy.

An acute infection with *C. burnetii* was defined as the presence of IgM antibodies against Ph2 antigen or/and a seroconversion. Raised IgG antibodies against Ph1 antigen of more than 1:800 were determined to be a serological profile of a chronic infection.

### Polymerase chain reaction

Two different PCR protocols were used. Both target the transposase of insertion sequence element IS 1111.

All placentas, amniotic fluids and colostrum/milk from patients exposed in the Soest outbreak were investigated using primer CoxP4 (5`TTAAGGTGGGCTGCGTGGTGATGG) and CoxM9 (5`GCTTCGTCCCGGTTCAACA ATTCG) according to the conventional PCR by Schrader [35]. It amplifies a 448-bp fragment of genomic DNA. The temperature regime was modified as touchdown PCR with five cycles of a declining annealing temperature (75 to 67°C in steps of 2°C). The DNA was extracted using the Puregene Extraction Kit (Biozym) as described by the manufacturer.

All placentas collected during the outbreak in Jena were investigated by a nested PCR according to Fenollar [2]. For the first amplification cycle the primers IS111 F1 (5`TACTGGGTGTTGATATTGC-3`) and IS111 R1 (5`-CCGTTTCATCCGCGGTG-3`), which target a 485-bp fragment were used. PCR was first carried out using the following profile: 95°C for 3 min, 95°C for 30 sec, 52°C for 30 sec, 72°C for 1 min (40 cycles), 72°C for 4 min. Reamplification was performed with the IS111 F2 (5`-GTAAAGTGATCTACACGA-3`) and IS 111 R2 (5`-TTAACAGCGCTTGAACGT-3`) primers, which target a 260-bp fragment: 95°C for 3 min, 95°C for 30 sec, 52°C for 30 sec and 72°C for 30 sec (30 cycles), 72°C for 4 min. The PCR-samples were analysed using gel electrophoresis.

### Culture

The placentas collected during the Soest outbreak were examined by cell culture using Buffalo Green Monkey (BGM) cells and a serum free-medium (UltraCulture, Bio Whittaker Europe, Verviers, Belgium) without antibiotics. The samples were homogenised using sterile sand and cell culture medium, the supernatants filtered through 0.2 μm syringe filters (Minisart, Sartorius, Hannover, Germany) and used as inocula. Cell cultures were incubated at 35°C and 5% CO_2_ and investigated weekly for seven weeks using phase contrast microscopy [36].

## Competing interests

The authors declare that they have no competing interests.

## Authors’ contributions

KB assembled the data, carried out the literature review and drafted the manuscript, AB took care of the women in Jena and made substantial contributions to interpretation of clinical data, CWW supervised both outbreaks and carried out the immunoassays as well as the PCR, BH contributed substantially including translation to the literature review, KH carried out the culture and PCR, TJ revised the manuscript and contributed significantly to its design, TS investigated the outbreak Jena and performed the follow up of the screened women, MB and ES helped draft the manuscript, DT collected the data of the outbreak Soest and drafted the manuscript. All authors read and approved the final manuscript.

## Pre-publication history

The pre-publication history for this paper can be accessed here:

http://www.biomedcentral.com/1471-2334/12/359/prepub
